# P-2105. Successful Implementation of Pediatric Infectious Diseases Interprofessional Consults for Multiple Patient Populations and Opportunities for Expansion

**DOI:** 10.1093/ofid/ofaf695.2269

**Published:** 2026-01-11

**Authors:** Sophie E Katz, Matthew Peworchik, Ritu Banerjee, C Buddy Creech, Walter Dehority, Daniel Dulek

**Affiliations:** Vanderbilt University Medical Center, Nashville, TN; Vanderbilt University Medical Center, Nashville, TN; Vanderbilt University Medical Center, Nashville, TN; Vanderbilt University Medical Center, Nashville, TN; Vanderbilt School of Medicine, TN; Vanderbilt University Medical Center, Nashville, TN

## Abstract

**Background:**

Curbside consults offer ease of communication but are inadequate for patient-level review and decision making. Peer-to-peer electronic consultations with a verbal component (interprofessional consults; IPCs) enable secure clinician communication, chart review, documentation, and billing in the electronic medical record (EMR). Prior studies show benefit of IPCs for inpatient teams. This study evaluated use of pediatric infectious diseases (PID) inpatient and outpatient IPCs at our medical center, characterized barriers to implementation, and identified opportunities to increase IPC volume and reimbursement.

**Methods:**

The PID IPC service was provided to 3 clinician groups: pediatric hospitalists practicing at a suburban community hospital (starting 4/2022), clinicians caring for solid organ transplant or oncology patients practicing at a quaternary academic medical center (starting 11/2023) and community pediatricians practicing in rural settings in TN (starting 3/2024). We reviewed demographic and clinical data electronically abstracted from the EMR. Social Vulnerability Index and Rural-Urban Commuting Area Codes were derived using patient zip codes. We informally surveyed PID clinicians to assess barriers to IPC. Payer reimbursement was assessed by encounter charges within the EMR. Chi square test was used for comparisons.

**Results:**

In total, 69 IPCs were completed during the study period (Figure 1). Median patient age was 3.2 years (Table 1a). Referrals were made for patients residing throughout TN (Figure 2). Compared to other providers, community hospitalists submitted IPCs for patients that were younger (Table 1a) and less likely to be socially vulnerable (Table 1b). The most common reason for consultation was gastroenteritis (Table 1b). Barriers to IPC completion included the need for patient/caregiver verbal consent, infrequent charge capture and challenges of workflow optimization during periods of low utilization.

**Conclusion:**

IPCs were implemented successfully for multiple patient populations. Opportunities to expand this service include targeted outreach to hospitals and clinics to increase utilization, modification of requirements for patient/caregiver consent prior to consultation, and working with payers to optimize reimbursement.

**Disclosures:**

Sophie E. Katz, MD MPH, Merck Manuals: Honoraria|Optum: Advisor/Consultant|Pfizer: Grant/Research Support Matthew Peworchik, MD, Pfizer: Grant/Research Support C. Buddy Creech, MD, MPH, AstraZeneca: Advisor/Consultant|GSK: DSMB|Merck: Advisor/Consultant|Moderna: Grant/Research Support|SanofiPasteur: Advisor/Consultant|TDCowen: Advisor/Consultant Walter Dehority, MD, MSc, Merck: Grant/Research Support|Moderna: Grant/Research Support Daniel Dulek, MD, Astellas Pharma: Advisor/Consultant|Eurofins-Viracor: Grant/Research Support

